# Next generation crop models: A modular approach to model early vegetative and reproductive development of the common bean (*Phaseolus vulgaris L*)

**DOI:** 10.1016/j.agsy.2016.10.010

**Published:** 2017-07

**Authors:** C. Hwang, M.J. Correll, S.A. Gezan, L. Zhang, M.S. Bhakta, C.E. Vallejos, K.J. Boote, J.A. Clavijo-Michelangeli, J.W. Jones

**Affiliations:** a Agricultural & Biological Engineering Dept., University of Florida, FL, USA; b School of Forest Resources & Conservation, University of Florida, FL, USA; c Horticultural Sciences Dept., University of Florida, FL, USA; d Agronomy Dept., University of Florida, FL, USA

**Keywords:** Gene-based crop model, G by E effects, Modular, Dynamic QTL effect model, Node addition rate, Time to first anthesis

## Abstract

The next generation of gene-based crop models offers the potential of predicting crop vegetative and reproductive development based on genotype and weather data as inputs. Here, we illustrate an approach for developing a dynamic modular gene-based model to simulate changes in main stem node numbers, time to first anthesis, and final node number on the main stem of common bean (*Phaseolus vulgaris L.*). In the modules, these crop characteristics are functions of relevant genes (quantitative trait loci (QTL)), the environment (E), and QTL × E interactions. The model was based on data from 187 recombinant inbred (RI) genotypes and the two parents grown at five sites (Citra, FL; Palmira, Colombia; Popayan, Colombia; Isabela Puerto Rico; and Prosper, North Dakota). The model consists of three dynamic QTL effect models for node addition rate (NAR, No. d^− 1^), daily rate of progress from emergence toward flowering (RF), and daily maximum main stem node number (MSNODmax), that were integrated to simulate main stem node number vs. time, and date of first flower using daily time steps. Model evaluation with genotypes not used in model development showed reliable predictions across all sites for time to first anthesis (R^2^ = 0.75) and main stem node numbers during the linear phase of node addition (R^2^ = 0.93), while prediction of the final main stem node number was less reliable (R^2^ = 0.27). The use of mixed-effects models to analyze multi-environment data from a wide range of genotypes holds considerable promise for assisting development of dynamic QTL effect models capable of simulating vegetative and reproductive development.

## Highlights

• A modular dynamic model was built for early vegetative growth.
• This model is based on three modules built from linear mixed effect models.
• Each module accounts for daily QTL, E, and QTL × E effects on traits.
• The model simulates main stem node growth and flowering time for bean.

## 1 Introduction

Tools that integrate genetic, environment and management information to predict crop performance in contrasting environments are needed to meet global food demands and assist plant breeders in designing new cultivars for increased yield (Hatfield and Walthall, 2015). Crop models are biophysical process-based simulation tools that predict crop growth and yield for a range of soil, climate, and management conditions. However, although they include empirically-derived parameters that allow them to simulate performance of different varieties, they still lack the integration of actual genetic information and thus are limited in their connection to plant genetics (Boote et al., 1996). Of note is that parameters termed “genetic coefficients” or “Genotype-Specific Parameters (GSPs)” that describe phenology, plant architecture (leaf area, number and plant dimensions), and biomass allocation in existing crop models are not yet linked to any gene(s). They do not take into account gene-by-environment (G × E) or G × G interactions at the level of individual processes that are considered in the models. This lack of genetic information within the crop models requires multi-environment experiments to estimate the GSP values when new cultivars (genotypes) are released. This process is time consuming, costly, and limits the utility of crop models in plant breeding programs and other practical applications. This omission of G and G × E information in crop models is not surprising since many of these models were developed before this type of information was known. However, these models do include environmental sensitivities of the traits that allow them to simulate dynamic growth and development processes under targeted environments. Therefore, a next step is to integrate genetic information (G and G × E) into models to predict a genotype's performance in a targeted environment.

The advances in genomics, phenomics (phenotyping), and computational technologies within the last decade have given scientists the unprecedented opportunity to understand the shaping of a given crop phenotype by the complex interactions among genotype, environment, and management. For example, new DNA sequencing technologies have increased the number of genetic markers for identifying genes associated with phenotypic traits. Large-scale phenotyping methods such as the use of unmanned aerial vehicles (UAVs), robotics, and sensor technologies are reducing costs and time for collecting field phenotype measurements. Also, new computational and statistical tools are rapidly advancing our ability to identify genes and environmental factors that affect crop traits. In spite of the technical advances and statistical sophistication of gene mapping approaches, few researchers have tackled the prediction of phenotypic vegetative and reproductive development of a genotype as affected by G, E, and G × E using daily (or shorter time step) environmental inputs. Nor do most studies reveal biological insights into the mechanisms of crop performance in specific environments (reviewed in Technow et al., 2015). Since crop models have the capacity to model daily vegetative and reproductive development from a mechanistic standpoint, and we can quantify some G × E interactions at a process level, integrating this information into crop models provides an opportunity to build the next generation of gene-based crop models.

The earliest and most common approach for integrating genetic information into crop models is linking specific genes to model parameters (i.e., the model's GSPs that had been estimated from field data, see White and Hoogenboom, 1996, White and Hoogenboom, 2003, and a more recent review by Yin et al., 2004). For example, GSPs in the CROPGRO-Soybean model were converted into mathematical functions of day length-sensitive genes (*E* loci), which were used to simulate the flowering and maturity behavior of soybean based on genetic information of cultivars (Messina et al., 2006). Chenu et al. (2009) modified the APSIM maize model (Keating et al., 2003) with parameters for leaf and silk elongation that were computed with equations that contained the quantitative trait loci (QTL). They were able to simulate a genotype's growth under drought conditions using this method. Others have shown that using whole-genome prediction methods (statistical approaches) when linked with crop models, have increased accuracy in prediction of yield in new environments in comparison with using statistical approaches alone (Technow et al., 2015). While these approaches appear to be promising methods for integrating genetics into crop models, current crop models lack specific gene-by-environment interactions at a process level and many models assume uniform environmental responses across genotypes.

QTL analyses can dissect the genetic architecture of complex traits, and in combination with statistical methods, such as mixed effect models, it is possible to estimate the genetic, environmental, and G × E effects on the phenotype (Boer et al., 2007, Chenu et al., 2009, Peiffer et al., 2014). We propose that these mixed effect approaches can be used to identify QTL, E, and QTL × E interactions underlying specific crop processes and that together with the decades of understanding of processes mechanisms from crops models can be combined to build a gene-based crop model that predicts aspects of crop performance based on genetic, environment, and management data. One advantage to this approach is that crop models already have subroutines (modules) that simulate different processes, such as phenological development, leaf area expansion, dry matter accumulation, and seed growth that are integrated together to simulate overall crop growth (Jones et al., 2001, Jones et al., 2003). Specific subroutines modeling selected biological processes could be modified to incorporate G, E, and G × E effects on those particular processes without having to alter other processes within the model (Boote et al., 2013). Also, studies have already demonstrated the usefulness of using this type of QTL-based approach to modify modules within crop models (Yin et al., 2000a, Reymond et al., 2003, Nakagawa et al., 2005, Messina et al., 2006, Uptmoor et al., 2008, Chenu et al., 2009).

The common bean (*Phaseolus vulgaris L.*) is the legume with the highest level of direct consumption around the world and is an important protein and nutrient source for the malnourished poor in Latin America and Africa (Broughton et al., 2003). The genome of the bean is relatively small with 11 chromosomes, and its sequence was recently published (Schmutz et al., 2014). Models of different crops have been developed from the early SOYGRO soybean model (Wilkerson et al., 1983), including the DSSAT CROPGRO-Bean model (Hoogenboom et al., 1994). These models provide established structure to explore different strategies to integrate genetic information or develop novel gene-based models. Due to the diversity in phenotypic responses and the well-established crop model for bean, it is a good test case for linking genetic information with a process-based model.

Here, we describe a prototype gene-based model that simulates the main stem node number over time and flowering date for common bean as affected by the genotype of the crop (represented by QTLs), and its response to the environment, and genotype-by-environment interactions (QTL × E) by integrating dynamic QTL effect models for daily development rate of progress toward appearance of the first flower (RF(*t*), modified from Bhakta, 2015), daily maximum main stem node number (MSNODmax), and, node addition rate (NAR(*t*), Zhang, 2015). The modules described here incorporate relationships (QTL, E, and QTL × E) previously identified through a linear mixed effect statistical model approach, knowledge of physiological processes, and a daily time step with corresponding E inputs that vary daily (e.g., day length, temperature, solar radiation). The described approach traces a path toward building a next generation of gene-based crop models using QTL, E, and QTL × E interaction effects on separate development and growth processes, which are hypothesized to be more capable of predicting the phenotype of specific genotypes over a range of environments.

## 2 Materials & methods

### 2.1 Plant materials & field sites

The details on the RI family of the common bean that were used in these studies can be found in Bhakta et al. (2015). Briefly, the RI family of 187 genotypes was generated from a cross between the determinate Andean cultivar, *Calima*, with an indeterminate Mesoamerican cultivar, *Jamapa*, for these studies (Bhakta^b^ 2015). The population was developed through single seed descent to the 11th generation, and bulked to the 14th generation (F_11:14_). The population was then planted across five field sites: Citra, FL (CT); Palmira, Colombia (PA); Popayan, Colombia (PO); Isabela, Puerto Rico (PR); and Prosper, North Dakota (ND). Details on the field sites are provided in Table 1 with the weather files in supplemental table (Table A.1 weather information). The experimental design followed a latinized, row-column design with three replicates (3 plots of each genotype, and 6 to 9 plots for each parent line). Details of the experiment design are presented in Clavijo (2015).

**Table 1:** Site management summary.

Site^a^	CT	PA	PR	PO	ND
Latitude	29 39′ N	03 29′ N	18 28′ N	02 25′ N	47 00′ N
Longitude	82 06′ W	76 81′ W	61 02′ W	76 62′ W	96 47′ W
Elevation^b^ (m)	60	1000	128	1800	280
Growing season	Mar, 2011 to	Nov, 2011 to	Feb, 2012 to	Mar, 2012 to	May 2012 to
	Jun. 2011	Jan, 2012	May 2012	Jun, 2012	Aug, 2012
Previous culture	Fallow	Beans	Beans	Fallow	Wheat
Soil texture	Sand	Clay	Clayey kaolinite	Medium loam	Silt/clay loam
Fertilization [kg ha^− 1^]	N-P-K:136-60-112	40 (Urea)	55(N-P-K:10-10-10)	N-P-K:129-96-80.3	No fertilizer
Irrigation	Cent pivot	Rain fed	Drip	Rain fed	Rain fed
Plant density [plans m^− 2^]	4.3	3	3.9	4.3	3.3
Row spacing [cm]	90	120	100	90	150
# of replicates	3	3	3	3	3
# of genotypes	168	174	128	178	176
TMEAN [°C] ^c^	24.61	23.94	24.36	18.08	20.39
TMAX [°C]^c^	31.87	28.80	25.41	29.24	11.93
TMIN [°C]^c^	17.93	19.49	14.07	20.49	− 1.63
SRAD [MJ m^− 2^ d^− 1^]^c^	20.41	14.67	15.78	22.58	15.01
DL [h d^− 1^]^c^	13.36	11.82	12.90	12.21	14.96

a Abbreviations of CT, PA, PR, PO, and ND represent corresponding sites of Citra, FL (CT); Palmira, Colombia (PA); Popayan, Colombia (PO); Isabela, Puerto Rico (PR); and Prosper, North Dakota (ND).

b Meters above sea level.

c Average environmental values taken over the season within each site.

### 2.2 Phenotyping

Two types of phenotypic data were collected. The first data were non-destructive measurements in which 6 plants per plot (marked after emergence) were observed every 2 to 3 days for developmental time-to events (such as time to first anthesis). For each replication in each plot, the number of days it took 50% of the plants to reach anthesis was determined. The second type of data included weekly destructive samplings of 3 replicates (one plant per plot) performed after emergence of the first true leaf. Samples were collected at each site depending on the availability of plants and measurements of node numbers were recorded for the main stem on each day of sampling. In this study, we examined the duration between emergence and first flower (TF), maximum node number on the main stem (MSNODmax), and number of nodes on the main stem on day *t* (N_obs_(*t*)) were used to model rate of progress toward flowering (RF), MSNODmax, and node addition rate (NAR) respectively. Of note, nodes after the unifoliate node position were counted for number of nodes on the main stem (i.e., number of trifoliate).

### 2.3 Molecular marker & linkage mapping

A SNP-based linkage map was constructed with the RI family using the genotyping-by-sequencing (GBS) method described in Bhakta et al. (2015). The final linkage map comprised 513 molecular markers with an average interlocus distance of 1.9 cM.

### 2.4 Training and evaluation dataset

The subsetting of data for model development and evaluation is described in Table 2. Modules for each of the three traits (RF, MSNODmax, and NAR) were developed with the same 171 RILs grown across the 5 sites, while evaluation of each module was performed with the remaining RILs as well as the 2 parents.

**Table 2:** Number of genotypes (RILs) used for model development (training) and model evaluation for trait modules, RF, MSNODmax and NAR.

	RF	MSNODmax	NAR
Training	171	171	171
Evaluation	16 + 2 parents	15 + 2 parents	9 + 2 parents

### 2.5 Linear mixed effect analysis

Boer et al. (2007) have described in detail procedures to develop statistical linear mixed effect models to evaluate, for a given phenotype, the magnitude of the G (QTL), E, and G (QTL) × E interactive effects on traits, which were implemented in this study using the training dataset of 171 RILs at the five sites. The procedures were based on multi-environment, single trait analysis with an unstructured error variance–covariance matrix. Static trait values were first fitted with the linear mixed effect model shown in Eq. (1)

(1) $$y_{\mathrm{ijk}}=\mu +{S_{i}+G}_{j}+S_{i}G_{j}+\varepsilon_{\mathrm{ijk}}$$

where a static phenotypic trait (*y*) is predicted with the trait general mean (μ); fixed site effect for site *i* (S_*i*_); random genetic effect for genotype *j* (G_*j*_); random G × S interactive effect (S_*i*_G_*j*_); and random error (ε_*ijk*_). Next, the genetic effect in Eq. (1) was expanded to include QTL effects to form Eq. (2).

(2) $$y_{\mathrm{ijk}}=\mu {+S_{i}+{\mathrm{QTL}}_{j,q}+G}_{j}+S_{i}{\mathrm{QTL}}_{j,q}+S_{i}G_{j}+\varepsilon_{\mathrm{ijk}}$$

This step identified the fixed QTL effects for marker region (*q*) in genotype (*j*) (*QTL*_*j* , *q*_) that were significant in explaining the random genetic effect in Eq. (1). Furthermore, Eq. (2) identified fixed site-by-QTL interaction effects (S_*i*_QTL_*j* , *q*_). The site effects in Eq. (2) were further expanded with environmental terms to give Eq. (3).

(3) $$y_{\mathrm{ijk}}=\mu +E_{i,e}{+S_{i}+{\mathrm{QTL}}_{j,q}+G}_{j}+E_{i,e}{\mathrm{QTL}}_{j,q}+S_{i}{\mathrm{QTL}}_{j,q}+S_{i}G_{j}+\varepsilon_{\mathrm{ijk}}$$

This step identified the fixed effects for environmental covariate *e* at site *i* (*E*_*i* , *e*_) that were significant in explaining fixed site effect in Eq. (2). Furthermore, Eq. (3) identified environmental covariates that were interacting with QTL regions (E_*i* , *e*_QTL_*j* , *q*_). The examined environmental covariates included: average temperature (TMEAN), minimum temperature (TMIN), maximum temperature (TMAX), average day time temperature (TD), average night time temperature (TN), average day-night temperature differences (TDN), solar radiation (SRAD), and day length (DL).

Accordingly, this methodology was applied to fit statistical models for the traits to identify significant QTL, E, and QTL × E factors for the selected processes in this study using GenStat 15th edition (Payne et al., 2009). The linear mixed effect model developed by Bhakta (2015) for time to flowering (TF) used genotypic and environmental covariates averaged over the time between emergence and first flower; the linear mixed effect model presented here for MSNODmax used genotypic and environmental covariates averaged over the time between emergence and first observation of final main stem node number; the linear mixed effect model developed by Zhang (2015) for NAR used genotypic and environmental covariates averaged over the duration of linear node addition.

### 2.6 Dynamic QTL effect model

Linear mixed effect models that estimate static trait values across season cannot simulate the dynamic behavior of key traits as they respond to changes in explanatory variables within the season. For example, explanatory variables such as temperature and SRAD fluctuate on daily or hourly basis. Therefore, dynamic models are needed. Information (i.e., QTL, E, and QTL × E terms affecting a trait) from Section 2.5 was used to guide the construction of dynamic QTL effect models. We simplified the approach for temperature responses by using daily mean temperature (TMEAN(*t*)) instead of hourly or minimum and maximum daily temperatures. The dynamic QTL effect RF module used daily values of the environmental variables to predict daily rate estimates; this is the approach used in the CROPGRO-Bean model (Jones et al., 2003) and most dynamic crop models. Similarly, we developed the dynamic QTL effect NAR module to respond to daily environmental variables. By integrating the daily rates, the dynamic rate models (RF and NAR) account for variations in QTL, E and QTL × E interactions over time, which is important for predicting crop development in the field. For example, we used daily mean temperature (TMEAN(*t*)) values, even though Bhakta (2015) found that the individual temperature covariates (average max and min temperatures) over an extended time period were more influential in affecting the rate of development toward flowering than mean temperature.

The general scheme of the dynamic QTL effects model for each trait (i.e., two daily development rate traits, RF(*t*) and NAR(*t*), and one static trait (final main stem node number, N_final_) estimated with the MSNOD_max_ module) is presented in Eq. (4) below.

(4) $$y\left(t\right)=\mu +\sum_{i=1}^{e}\left\{b_{1_{i}}\cdot \left[E_{i}\left(t\right)-{\overset{-}{E}}_{i}\right]\right\}+\sum_{j=1}^{q}\left\{b_{2_{j}}\cdot {\mathrm{QTL}}_{j}\right\}+\sum_{i=1}^{e}\sum_{j=1}^{q}\left\{b_{3_{i,j}}\cdot {\mathrm{QTL}}_{j}\cdot \left[E_{i}\left(t\right)-{\overset{-}{E}}_{i}\right]\right\}+\varepsilon_{\mathrm{ijk}}$$

where a phenotypic trait (*y*(*t*)) for a day (*t*) is predicted with the trait general mean (μ); trait environment effect parameter (*b*_1_*i*__) for the i^th^ environment covariate (ECV); daily ECV values (E_i_(t)); ECV means across sites for a trait $\left({\overset{-}{E}}_{i}\right)$; trait QTL effect parameters (*b*_2_*j*__) for the j^th^ QTL marker; QTL marker values (QTL_j_); trait QTL × E parameters (*b*_3_*i* , *j*__); and trait QTL × E effects $\left({\mathrm{QTL}}_{j}\cdot \left[E_{i}\left(t\right)-{\overset{-}{E}}_{i}\right]\right)$. Each dynamic QTL effect model (RF(*t*) and NAR(*t*)) thus uses daily environmental inputs with the equation structure shown in Eq. (4) whereas the MSNODmax module uses average environmental inputs over time from planting to the current simulation day (*t*) until anthesis to predict final number of nodes on the main stem.

### 2.7 Gene-based common bean model framework

**Fig. 1:**
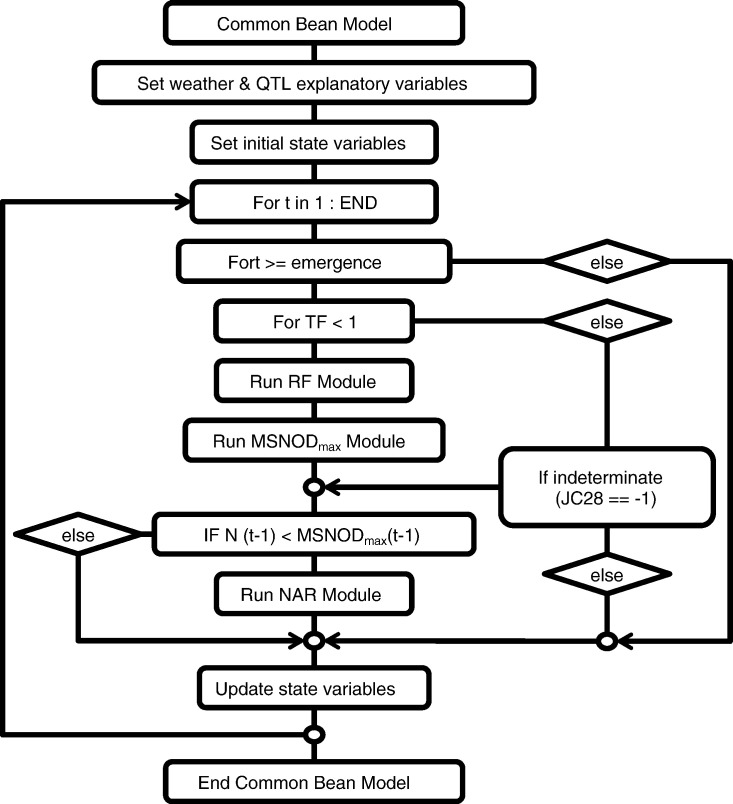
The framework of the gene-based Common Bean Model (GB-CBM) with the modules of rate of progress from emergence to flowering (RF), maximum node number on the main stem (MSNOD_max_), and main stem node addition rate (NAR). The input files include the daily weather and genotype information. Emergence days after planting and last day of experiment for a location (END) set simulation run time for a genotype at a site. Time to flowering (TF) is the state variable that accounts for phenology (anthesis). JC28 is the QTL region in the model that defines determinacy of a genotype for this model.

A prototype, gene-based Common Bean Model (GB-CBM) was developed by combining the three modules that simulate RF, MSNODmax, and NAR and integrating the daily-predicted rates to simulate day of first flower, node numbers on the main stem over time, and final number of main stem nodes for common bean. The structural layout of the GB-CBM is presented in Fig. 1. Input files for weather, management, field observations, genetic marker information (QTL), and gene-based module parameter values are read at program initiation. A genotype in the population is selected and simulated for each of the five sites: Citra, FL (CT); Fargo, ND (ND), Palmira, Colombia (PA); Popayan, Colombia (PO); and Isabela, Puerto Rico (PR). For each genotype at a site, variables are computed based on daily weather information from the start (planting) to the last day of the experiment (DAY). The GB-CBM then runs the modules for flowering ((duration to flowering)^− 1^, RF), maximum main stem node number (MSNODmax), and main stem node addition rate (NAR) to simulate the dynamic changes in main stem node number and time to first flower for each genotype at a site. This process is repeated for the entire population to produce a simulated distribution of main stem node numbers over time, days to first flower across multiple sites, and final node number.

The RF(*t*) and NAR(*t*) rate modules were run each simulation day based on the allelic makeup at relevant QTLs and the daily environmental conditions for each RI line and across all five environments. The day of first flower was simulated for each line growing in each environment by the computed development progress, TF(*t*). TF(*t*) was computed by numerically integrating daily computations of RF(*t*), starting at emergence (Eq. (5)).

(5) $$\mathrm{TF}\left(t\right)=\mathrm{TF}\left(t-1\right)+\mathrm{RF}\left(t\right)\cdot \mathrm{dt},\;\mathrm{for}\;\left(\text{emergence date}\;\leq \;t\mathrm{and}\;\mathrm{TF}\;<\;1\right)$$

Simulated nodes on each day (N(*t*)) were obtained by numerical integration of the NAR values as follows:

(6) $$\begin{array}{c} N\left(t\right)=N\left(t-1\right)+\mathrm{NAR}\left(t\right)\cdot \mathrm{dt}\;\mathrm{for}\;\left(\mathrm{TF}\;<\;1\right)\;\mathrm{for}\;\text{determinate genotypes}\;\\ \;\text{or}\left(\mathrm{any}\;\mathrm{TF}\;\mathrm{for}\;\text{indeterminate genotypes}\right)\\ \;\mathrm{and}\;\left(N\left(t-1\right)\;<\;\text{MSNODmax}\left(t-1\right),\mathrm{for}\;\mathrm{all}\;\text{genotype}\right) \end{array}$$

where, the simulation starts at time *t* equal to or greater than emergence day. For all genotypes, initial node number is set at 0.0 at *t* equal to emergence day*.* For determinate genotypes, numerical integration with NAR(*t*) is performed as long as first flower has not occurred and node number is less than the maximum main stem node number (N_final_). For indeterminate genotypes, numerical integration with NAR(*t*) is performed beyond first flower and while node number is less than the final main stem node number (N_final_). *dt* is the numerical integration time step (*dt* = 1 day in this study). For determinate genotypes, final main stem node number (N_final_) is set at first flower or when node number reaches the maximum main stem node number estimate of the previous day. For indeterminate genotypes, final main stem node number (N_final_) is set when node number reaches the estimate of maximum main stem node number estimate of the previous day (MSNODmax(t − 1)).

In the GB-CBM, the QTL (i.e., JC28) region that contains the *FIN/TFL1Y* gene defines the determinacy of a genotype (Repinski et al., 2012). That QTL region determines whether a terminal inflorescence will develop on the main stem. Due to the bi-parental nature of our RI population, *Calima* (determinate) markers are assigned the value of + 1 while *Jamapa* (indeterminate) markers are assigned − 1. Therefore, a genotype is indeterminate and continues to add nodes up to the maximum main stem node number after flowering if it has a QTL marker value of − 1 for that region, otherwise node addition on the main stem ends on the day of first flower appearance (i.e., determinate genotypes stop main stem node addition at anthesis). Molecular marker data used in the models are provided in supplemental table, Table A.2 Genotype. In contrast to the daily development rate traits (NAR(*t*) and RF(*t*)), the MSNODmax module was initiated at emergence for each genotype at a given site, but used average environment values from planting to current simulation day (*t*) to estimate daily maximum main stem node numbers. The module was terminated upon simulated anthesis and maximum main stem node numbers were set. Further studies are needed to determine the exact window for the environment as they affect apical node development and differentiation. Parameters for each dynamic QTL effect model were estimated for daily time steps described in Section 2.8.

### 2.8 Model calibration & evaluation

All modules were built using the R programming language (version 3.2.3; R Core Team) and the dynamic QTL effect model for each module was calibrated with the training set of 171 RILs across the 5 sites using the nonlinear least squares algorithm in the minpack.lm package (Elzhov et al., 2013) as implemented in the statistical package R to estimate parameters for the explanatory terms in Eq. (4) for each of the three traits modeled in this study. Initial values for each parameter were those identified from the linear mixed effect model for each process. Because we are using dynamic models instead of simple static equations to model node number and time of first flower, we used the approach described by Wallach, (2006) to numerically simulate values using QTL, E, and parameter inputs for comparison with each observed value to compute errors. To estimate the parameters associated with the QTL and E components of the model for RF(*t*), errors between observed and simulated duration between emergence and first flower, across all environments (*S*) and genotypes (*G*), were used to estimate parameters of the dynamic QTL effect model for first flowering (Eq. (7)).

(7) $$\mathrm{SSQError}\left(\mathrm{FL}\right)=\sum_{S=1}^{5}\sum_{G=1}^{171}{\left(\mathrm{FL}-{\mathrm{FL}}_{\mathrm{obs}}\right)}^{2}$$

where FL is simulated day of first flower and FL_obs_ is observed first flower day. Similar to the criterion for fitting the RF(*t*) dynamic model, the sum of square error between observed number of nodes measured on each date during the linear phase of node addition (N_obs_(*t*)) and simulated number of nodes on those same dates (N(*t*) were used to estimate the parameters of the dynamic model for node addition rate that minimizes the sum of squared errors (SSQError(N)) between observed and simulated node numbers across all environments (S), genotypes (G), and observation dates (*t*) (Eq. (8)).

(8) $$\mathrm{SSQError}\left(N\right)=\sum_{S=1}^{5}\sum_{G=1}^{171}\sum_{1}^{\mathrm{obs}}{\left(N\left(t\right)-N_{\mathrm{obs}}\left(t\right)\right)}^{2}$$

The MSNODmax module parameters were estimated based on minimizing the sum of squared errors (SSQError(N_final_)) between observed (N_final,obs_) and simulated maximum node numbers (N_final_) across all environments (S), and genotypes (G) (Eq. [9]).

(9) $$\mathrm{SSQError}\left(N_{\mathrm{final}}\right)=\sum_{S=1}^{5}\sum_{G=1}^{171}{\left(N_{\mathrm{final}}-N_{\mathrm{final},\mathrm{obs}}\right)}^{2}$$

where N_final_ is simulated final node number determined by using average environment values from planting to first observed final node number for each genotype at a site.

Model evaluations using the evaluation set of genotypes were performed with the two common bean parents as well as an additional 16 RI genotypes for RF(*t*), 15 for MSNODmax, and 9 RI genotypes for NAR(*t*) using R^2^, %RMSE, %Bias, and d-statistics (Willmott et al., 1985).

## 3 Results

### 3.1 Linear mixed effect analysis

**Fig. 2:**
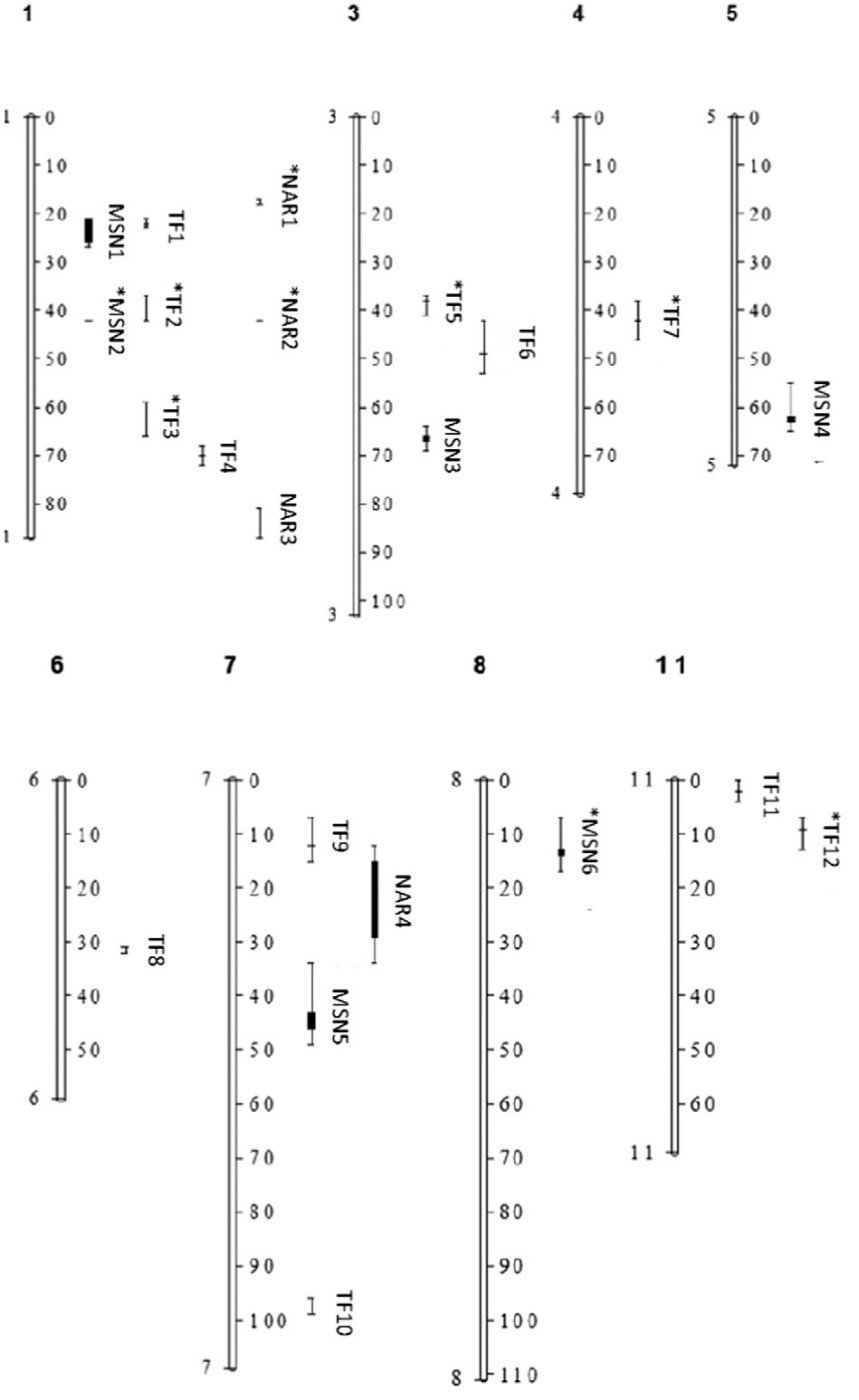
The QTL that were identified from multi-environment composite interval QTL mapping for time from emergence to flowering (TF), maximum number of nodes along the main stem (MSNOD_max_), and node addition rate (NAR) in the common bean RI population. TF*i* are markers (Bhakta, 2015) that were used in the RF module, MSN*i* are markers that were used in the MSNOD_max_ module, and *NARi* are markers that were used in the NAR module (Zhang, 2015). Markers with QTL × E are denoted with the * symbol. Bars denote the 1 LOD intervals while whiskers denote the 2 LOD intervals from the peak LOD value for each identified QLT marker.

The linear mixed effect models for the RF, MSNODmax, and NAR traits identified a total of 22 QTLs with 10 of these having QTL × E interactions. The QTLs for each module were designated as TF*i* for QTLs found to affect time from emergence to first flower (within the RF module), MSN*i* for QTLs found to affect maximum main stem node number (within the MSNODmax module), and *NARi* for QTLs found to influence node addition rate (within the NAR module), with *i* denoting the order which the QTLs were named (Fig. 2). All E, QTL, and QTL × E interactive terms in the linear mixed effect models had chi-square *P*-values < 0.05. The chi-square probability for terms in the MSNODmax linear mixed effect model is reported in supplemental Table A.3. Subsets of QTLs associated with each of the three traits were found on the same chromosome segment. For example, QTLs TF2, MSN2, and NAR2, were found in the same region of chromosome 1. Given the fact that recombination is significantly suppressed in this region, it is highly unlikely that these QTLs will be easily resolved by recombinational analysis. However, we must point out that these QTLs are in the same region occupied by *FIN/TFL1Y*, a gene that has been found to control growth habit and therefore affect main stem node number (Repinski et al., 2012). Accordingly, genotypes with TF2, MSN2, and NAR2 with value + 1 were determinate and stopped main stem node addition at first flower, while indeterminate genotypes (TF2, MSN2, and NAR2 with value − 1) continued node addition up to the maximum node number after anthesis. Additional experimentation and molecular analyses will need to be performed to confirm the role of these QTLs/genes and environmental covariates in node development and time to first flower, and to extend the model for QTL × QTL interactions.

### 3.2 Dynamic QTL effect modules

The dynamic QTL effect model for RF(*t*) is shown below (Eq. (10)) with estimated parameter values for the dynamic, daily model. Temperature and day length interactions have been shown to affect flowering time for common bean by White and Kornegay (1994) and were included in this analysis but not in the liner mixed effect model presented by Bhakta (2015). The parameter IDs, estimated parameter values, and standard errors (SE) for the RF module are reported in Table 3.

(10) $$\begin{array}{c} \mathrm{RF}\left(t\right)=0.029+7.5\cdot 10^{-4}\left(\mathrm{TMEAN}\left(t\right)-21.35\right)-7.3\cdot 10^{-6}\left(\mathrm{SRAD}\left(t\right)-18.31\right)-2.2\cdot 10^{-3}\left(\mathrm{DL}\left(t\right)-12.7\right)\\ -3.3\cdot 10^{-4}\left(\mathrm{TMEAN}\left(t\right)-21.35\right)\left(\mathrm{DL}\left(t\right)-12.7\right)\\ +9.8\cdot 10^{-4}\cdot \mathrm{TF}1+1.7\cdot 10^{-3}\cdot \mathrm{TF}2-3.9\cdot 10^{-4}\cdot \mathrm{TF}3+2.0\cdot 10^{-4}\cdot \mathrm{TF}4\\ -1.5\cdot 10^{-4}\cdot \mathrm{TF}5+8.9\cdot 10^{-4}\cdot \mathrm{TF}6-5.3\cdot 10^{-4}\cdot \mathrm{TF}7-3.1\cdot 10^{-4}\cdot \mathrm{TF}8\\ -3.4\cdot 10^{-4}\cdot \mathrm{TF}9-9.7\cdot 10^{-5}\cdot \mathrm{TF}10+2.6\cdot 10^{-4}\cdot \mathrm{TF}11-6.6\cdot 10^{-5}\cdot \mathrm{TF}12\\ +\mathrm{TF}2\left(-3.6\cdot 10^{-5}\left(\mathrm{TMEAN}\left(t\right)-21.35.\right)\right)\\ +\mathrm{TF}3\left(6.7\cdot 10^{-5}\left(\mathrm{TMEAN}\left(t\right)-21.35\right)-1.1\cdot 10^{-3}\left(\mathrm{DL}\left(t\right)-12.7\right)\right)\\ +\mathrm{TF}5\left(5.5\cdot 10^{-5}\left(\mathrm{TMEAN}\left(t\right)-21.35\right)\right)\\ +\mathrm{TF}7\left(-2.6\cdot 10^{-4}\left(\mathrm{DL}\left(t\right)-12.7\right)\right)\\ +\mathrm{TF}12\left(-6.4\cdot 10^{-6}\left(\mathrm{SRAD}\left(t\right)-18.31\right)-3.9\cdot 10^{-4}\left(\mathrm{DL}\left(t\right)-12.7\right)\right) \end{array}$$

**Table 3:** The terms in the dynamic QTL effect model showing the parameter IDs and estimated parameter values with standard errors (SE) for 1/duration from emergence to flowering (RF).

Term	Parameter ID	Estimated value (SE)^b^
Mean RF	RFb	0.029 (1.5E − 4)
TMEAN^a^	RF_b1.1_	7.5E − 4 (3.6E − 5)
SRAD^a^	RF_b1.2_	− 7.3E − 6 (1.4E − 5)
DL^a^	RF _b1.3_	− 2.2E − 3 (8.9E − 5)
TMEAN × DL^c^	RF _b1.4_	− 3.3E − 4 (2.3E − 5)
TF1	RF _b2.1_	9.8E − 4 (1.1E − 4)
TF2	RF _b2.2_	1.7E − 3 (1.3E − 4)
TF3	RF _b2.3_	− 3.9E − 4 (1.5E − 4)
TF4	RF_b2.4_	2.0E − 4 (1.3E − 4)
TF5	RF_b2.5_	− 1.5E − 4 (1.2E − 4)
TF6	RF_b2.6_	8.9E − 4 (1.2E − 4)
TF7	RF_b2.7_	− 5.3E − 4 (9.9E − 5)
TF8	RF_b2.8_	− 3.1E − 4 (8.9E − 5)
TF9	RF_b2.9_	− 3.4E − 4 (9.0E − 5)
TF10	RF_b2.10_	− 9.7E − 5(9.0E − 5)
TF11	RF_b2.11_	2.6E − 4 (1.5E − 4)
TF12	RF_b2.12_	− 6.6E − 5 (1.5E − 4)
TF2 × TMEAN^a^	RF_b3.1_	− 3.6E − 5 (3.4E − 5)
TF3 × TMEAN^a^	RF _b3.2_	6.7E − 5 (3.7E − 5)
TF3 × DL^a^	RF _b3.3_	− 1.1E − 3 (7.1E − 5)
TF5 × TMEAN^a^ TMEAN^a^	RF _b3.4_	5.5E − 5 (2.6E − 5)
TF7 × DL^a^	RF _b3.5_	− 2.6E − 4 (5.9E − 5)
TF12 × SRAD^a^	RF _b3.6_	− 6.4E − 6 (1.3E − 5)
TF12 × DL^a^	RF _b3.7_	− 3.9E − 4 (5.8E − 5)

a Mean values across sites for TMEAN[°C]: SRAD[MJ·d^− 1^]: DL[*hr*] are 21.35:18.31:12.7, respectively.

b Estimated values are attained from non-linear least squares algorithm.

c TMEAN × DL was the only term not included in the original linear mixed effect model developed by Bhakta, 2015.

The first term on the right hand side of Eq. (10) is the overall average rate of progress toward flowering across sites. The value of 0.029 d^− 1^ indicates that on average, the time between emergence and first flower across all genotypes and sites was 34.5 days. The 4th term indicates that an hour increase above 12.7 h in day length would result in a 2.2E − 3 lower rate of development from the general mean of 0.029 rate of daily progress toward first flower. Increasing the day length by one hour will increase the time to first flower from 34.5 to 37.3 days provided all other variables were kept at their average values. This timing will also vary as a function of QTL alleles and their interactions with specific environmental variables as indicated in Eq. (10). This effect is analogous to the photoperiod sensitivity (PPSEN) parameter currently used in the DSSAT CROPGRO-Bean model to simulate development rate toward anthesis as affected by photoperiod. The *Calima* QTL allele, TF2^Cal^, will have a (+ 1) coefficient and therefore would increase the daily rate by a factor of 1.7E − 3 from the general mean rate of 0.029 toward first flower as a result of that QTL effect. Similarly, the same QTL allele will decrease the rate by a factor of 3.6E − 5 for a one degree increase in temperature above 21.35 °C. In contrast, the *Jamapa* allele, TF2^Jam^, will have the opposite effect. The sensitivity of the RF module to environmental factors can be seen in supplemental figure, Fig. A.1. However, not all *Calima* alleles affect the time to first anthesis in the same direction. For instance, although TF3^Cal^ will have a (+ 1) coefficient, the parameter value (− 6.0E − 4) of this QTL is negative indicating that the *Calima* allele of TF3 actually decreases the rate in contrast to the TF2^Cal^ effect.

Next, we present the parameters and equation developed for the MSNODmax module (Eq. (11)). The parameter IDs, estimated parameter values, and standard errors (SE) for the MSNODmax module are reported in Table 4.

(11) $$\mathrm{MSNODmax}\left(t\right)=12.37+0.43\left(\mathrm{TMEAN}\left(0:t\right)-21.85\right)+0.10\left(\mathrm{SRAD}\left(0:t\right)-18.74\right)+1.2\left(\mathrm{DL}\left(0:t\right)-12.81\right)-0.43\cdot \mathrm{MSN}1-3.56\cdot \mathrm{MSN}2-0.63\cdot \mathrm{MSN}3-0.20\cdot \mathrm{MSN}4-0.60\cdot \mathrm{MSN}5+0.32\cdot \mathrm{MSN}6\;+\mathrm{MSN}2\left[-0.08\left(\mathrm{TMEAN}\left(0:t\right)-21.85\right)-0.05\left(\mathrm{SRAD}\left(0:t\right)-18.74\right)-0.62\left(\mathrm{DL}\left(0:t\right)-12.81\right)\right]+\mathrm{MSN}6\cdot \left[-0.02\left(\mathrm{TMEAN}\left(0:t\right)-21.85\right)+0.01\left(\mathrm{SRAD}\left(0:t\right)-18.74\right)\right]$$

**Table 4:** The terms in the dynamic QTL effect model showing the parameter IDs and estimated parameter values with standard errors (SE) for maximum number of nodes along the main stem (MSNODmax) module.

Term	Parameter ID	Estimated value (SE)^b^
Mean MSNODmax	MSNODmax_b_	12.37 (0.13)
TMEAN^a^	MSNODmax_b1.1_	0.43 (0.05)
SRAD^a^	MSNODmax_b1.2_	0.10 (0.03)
DL^a^	MSNODmax_b1.3_	1.2 (0.08)
MSN1	MSNODmax_b2.1_	− 0.43 (0.12)
MSN2	MSNODmax_b2.2_	− 3.56 (0.15)
MSN3	MSNODmax_b2.3_	− 0.63 (0.10)
MSN4	MSNODmax_b2.4_	− 0.20 (0.10)
MSN5	MSNODmax_b2.5_	− 0.60 (0.10)
MSN6	MSNODmax_b2.6_	0.32 (0.12)
MSN2 × TMEAN^a^	MSNODmax_b3.1_	− 0.08 (0.05)
MSN2 × SRAD^a^	MSNODmax_b3.2_	− 0.05 (0.04)
MSN2 × DL^a^	MSNODmax_b3.3_	− 0.62 (0.09)
MSN6 × TMEAN^a^	MSNODmax_b3.4_	− 0.02 (0.05)
MSN6 × SRAD^a^	MSNODmax_b3.5_	0.01(0.03)

a Mean values across sites for TMEAN[°C]: SRAD[MJ·d^− 1^]: DL[*hr*] are 21.85:18.74:12.81, respectively.

b Estimated values are attained from non-linear least squares algorithm.

where TMEAN(0:*t*) is daily average temperatures averaged between planting (*t* = 0) and the current simulation day (*t*) up to simulated anthesis. Eq. (11) indicates that a one degree increase above 21.85 °C in the mean temperature would result in additional 0.43 nodes from the general mean of 12.37 maximum nodes as a result of the temperature effect. The *Calima* QTL allele, MSN2^Cal^, will have a (+ 1) coefficient and therefore would decrease the maximum node number by 3.56 from the general mean of 12.37 maximum nodes as a result of the QTL effect. Similarly, the same QTL allele will decrease the maximum nodes by 0.08 nodes for a one degree increase in temperature above 21.85 °C. In contrast, the *Jamapa* allele, MSN2^Jam^, will have the opposite effect.

The model for NAR(*t*) is shown below (Eq. (12)) with estimated parameter values for the dynamic model. The parameter IDs, calibrated parameter values, and standard errors (SE) for the NAR module are reported in Table 5.

(12) $$\begin{array}{l} \mathrm{NAR}\left(t\right)=0.252+2.0\cdot 10^{-2}\left(\mathrm{TMEAN}\left(t\right)-21.51\right)-7.9\cdot 10^{-4}\left(\mathrm{SRAD}\left(t\right)-17.38\right)+4.4\cdot 10^{-3}\left(\mathrm{DL}\left(t\right)-12.74\right)\\ \;-6.0\cdot 10^{-3}\cdot \mathrm{NAR}1+7.0\cdot 10^{-3}\cdot \mathrm{NAR}2+8.2\cdot 10^{-3}\cdot \mathrm{NAR}3-4.5\cdot 10^{-3}\cdot \mathrm{NAR}4\\ \;+\mathrm{NAR}1\cdot \left[-1.9\cdot 10^{-4}\left(\mathrm{DL}\left(t\right)-12.74\right)\right]\\ +\mathrm{NAR}2\cdot \left[2.1\cdot 10^{-3}\left(\mathrm{TMEAN}\left(t\right)-21.51\right)\right]\; \end{array}$$

**Table 5:** The terms in the dynamic QTL effect model showing the parameter IDs and estimated parameter values with standard errors (SE) for main stem node addition rate (NAR).

Term	Parameter ID	Estimated value (SE)^b^
Mean NAR	NARb	0.252 (4E − 3)
TMEAN^a^	NARb1.1	2.0E − 2 (5.7E − 4)
SRAD^a^	NARb1.2	− 7.9E − 4 (3.2E − 4)
DL^a^	NARb1.3	4.4E − 3 (8.0E − 4)
NAR1	NARb2.1	− 6.0E − 3 (1.0E − 3)
NAR2	NARb2.2	7.0E − 3 (1.0E − 3)
NAR3	NARb2.3	8.2E − 3 (9.2E − 4)
NAR4	NARb2.4	− 4.5E − 3 (9.0E − 4)
NAR1 × DL^a^	NARb3.1	− 1.9E − 4 (6.6E − 4)
NAR2 × TMEAN^a^	NARb3.2	2.1E − 3 (5.2E − 4)

a Mean values across sites for TMEAN[°C]: SRAD[MJ·d^− 1^]: DL[*hr*] are 21.51:17.38:12.74, respectively.

b Estimated values are attained from non-linear least squares algorithm.

where TMEAN(*t*) is the daily average temperature for simulation day (*t*). The average rate of node appearance in the study of 0.252 indicates that there was 3.97 days between the appearances of successive leaf tips. Eq. (12) indicates that a one degree increase above 21.51 °C in the temperature for a day would result in a 0.02 faster daily rate from the general mean of 0.252 rate of node addition per day as a result of the temperature effect. This linear temperature response is analogous to temperature response functions in existing DSSAT CROPGRO-Bean model, where cultivar development rate is calculated with non-linear, piecewise temperature response function *f*(T_base_, T_opt_). The *Calima* QTL allele, NAR2^Cal^, will have a + 1 QTL value and therefore would increase the rate of node appearance by 7.0E − 3 from the general mean of 0.252 nodes per day as a result of the QTL effect. Similarly, the same QTL allele will increase the node addition rate by 2.1E − 3 nodes per day for a one degree increase in temperature above 21.51 °C. In contrast, the *Jamapa* allele, NAR2^Jam^, will have the opposite effect. The sensitivity of the NAR module to environmental factors can be seen in supplemental figure, Fig. A.2.

### 3.3 Evaluation of GB-CBM

**Fig. 3:**
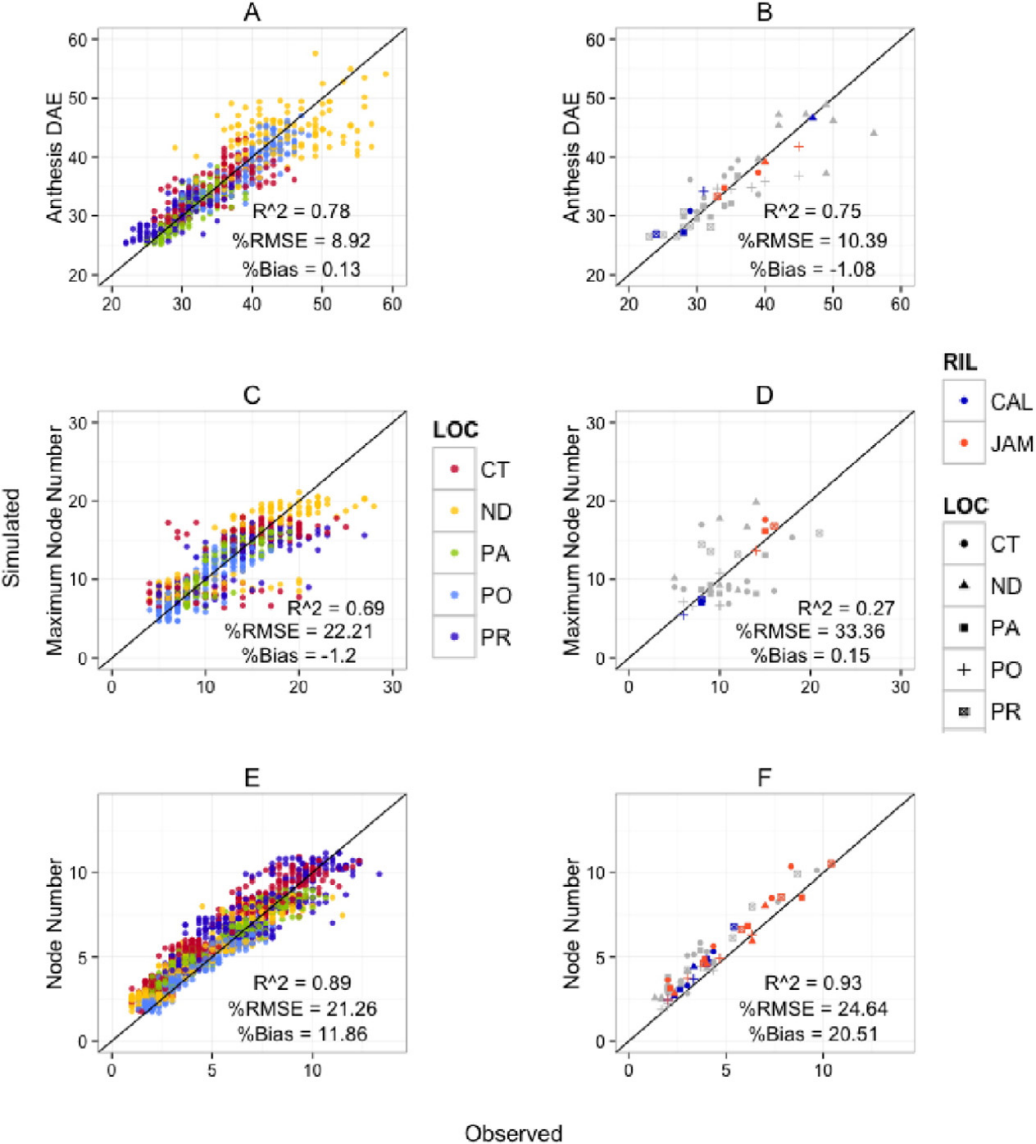
The simulated with daily time steps from the RF (anthesis days after emergence) (A and B), MSNOD_max_ (maximum main stem node number) (C and D), and node number during the linear phase of node addition predicted by NAR (node addition rate) (E and F) module versus observed data with 1:1 lines are shown for the calibration set (A, C, E) and evaluation set (B, D, F) across the five sites (CT, ND, PA, PO, PR). The evaluation set included the parents, *Calima* (CAL) in blue and *Jamapa* (Jam) in orange and RILs in grey with 14 additional lines for RF and MSNODmax, and 7 additional lines for NAR. The analyses of these plots included R^2^, %RMSE, and %Bias.

The RF module operating on daily time steps was able to capture the delay in flowering that was observed in ND (Fig. 3 A, B) since the dynamic QTL effect model had day length (DL), temperature × DL interaction (TMEAN × DL), and QTL × DL interaction terms. The RF module had an evaluation of R^2^, %RMSE, and %Bias values of 0.75, 10.4, and − 1.1, respectively across locations (Fig. 3 B). The MSNOD_max_ module did not perform as well as the other two modules for the evaluation set (R^2^, %RMSE, and %Bias values of 0.27, 33.36, and 0.15, respectively across locations; Fig. 3 D). It should be noted that a 0.15% bias would only result in over simulation of about 1.0 node or less. Our assumption for using ECV values over time periods of planting to current simulation day up to simulated anthesis to predict maximum main stem node number requires additional studies. There are likely additional environmental covariates that are affecting the variation in maximum main stem node number that were not considered. A source and sink relationship between photosynthesis and assimilate allocation could also be a driving force behind the final main stem node number. The few replications used (*n* = 3), frequency of measurements for each genotype (a sample per week), and sampling with different plants limit all of these analyses as well. The NAR module predicted node number over the linear phase of node addition well for the evaluation set but with high bias (R^2^, %RMSE, and %Bias values of 0.93, 24.64, and 20.51, respectively across locations; Fig. 3 F). The somewhat high bias in predicted node number (N) is partly due a propagation of error since predicted rates (each with some bias) were integrated for each day over the course of the simulation. Based on intercept values from a node addition rate linear regression analyses, time of first node appearance is likely genotype-by-environment specific. Therefore, an additional module should be added for the duration from emergence to appearance of first node for improved node development simulations.

**Fig. 4:**
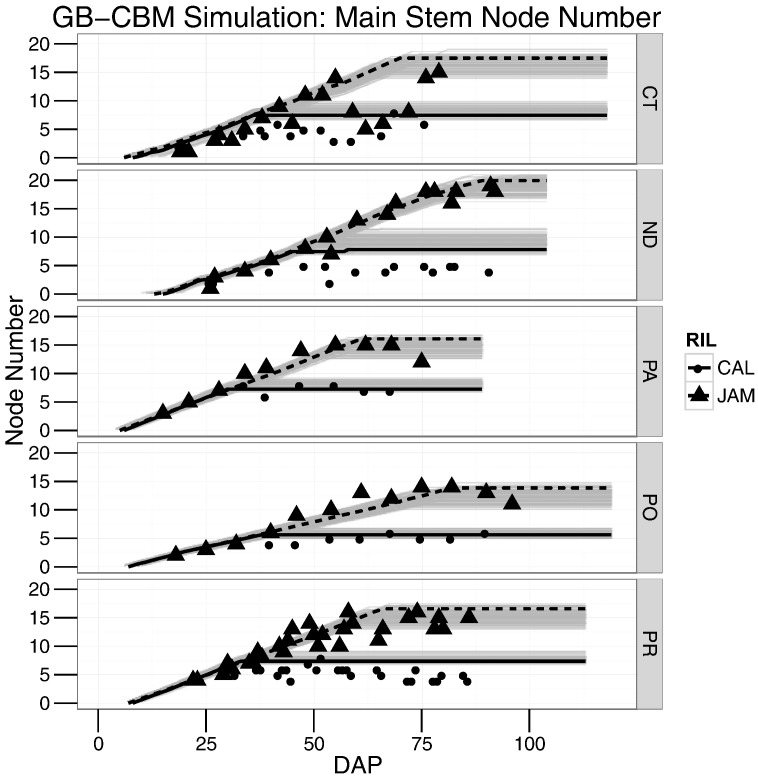
Simulation results using the GB-CBM to predict main stem node number over time for 187 genotypes (RILs), across the five field sites (CT, Citra, ND, North Dakota, PA, Palmira, PO, Popayan, PR, Puerto Rico) with observed data for the parents *Jamapa* (JAM) in triangle and *Calima* (CAL) in circles. The grey lines represent the RILs with them segregating based on JC28 QTL.

Integrating the three modules together for the GB-CBM (Fig. 1) provides the time series simulation for main stem node numbers for all 187 genotypes across the five sites (Fig. 4). The emergence of plants was delayed in ND and can be seen relative to the other sites (Fig. 4 ND vs. CT, PA, PO, PR). For determinate genotypes such as *Calima*, either simulated first flower or maximum main stem node number stopped the addition of main stem nodes. For indeterminate genotypes such as *Jamapa*, the MSNODmax module set the maximum main stem node number. The separation of the two groups can clearly be seen and is due to the strong effect of the QTLs called TF2, MSN2, and NAR2. These QTLs either represent the action of the *TFL1Y/FIN* gene, or the action of separate genes tightly linked to *FIN* (Fig. 4, grey lines). Of note, the range of grey lines represents the simulated main stem node numbers of the RIL population as they differed from the parents. The time series plots for node number showed the observed data from both *Calima* and *Jamapa* in CT, in which there were fewer node numbers at later time points, likely due to the extreme temperatures that may have caused failures in node formation for single observed plants sampled (Fig. 4, CT). This lower node number was associated with a large range of phenotypic responses found in CT (e.g., increased number of branches).

**Fig. 5:**
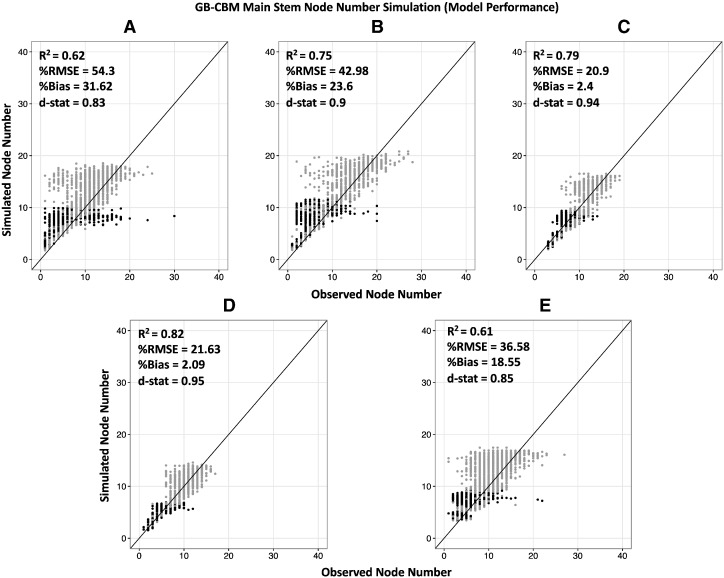
Simulated versus observed plots of main stem node number using the GB-CBM over the season for 187 genotypes (RILs) by field sites (Citra (A), North Dakota (B), Palmira (C), Popayan (D), Puerto Rico (E)) with R^2^, %RMSE, %Bias, and Willmot agreement index. Determinate genotypes are black, while indeterminate genotypes are grey. The parameters were estimated across all sites but plotted to compare performance at each site. The lines represent 1:1 relationships.

A comparison of the GB-CBM simulation results for node number across sites with all of the observed data over the season from the 187 RILs shows that the GB-CBM model had fairly good predictions of node number with an average across sites R^2^, %RMSE, %Bias, and Willmot agreement index of 0.72, 35.28, 15.65, and 0.89, respectively (Fig. 5). The overall GB-CBM node number simulation performance was reduced by CT and PR results with R^2^, %RMSE, %Bias, and Willmot agreement index of 0.62, 54.3, 31.62, and 0.83, respectively in CT, and 0.61, 36.58, 18.55, and 0.85, respectively in PR. Crops in CT experienced several days of hot temperatures and thus had a greater variability in their node number compared to other sites PO and PA (Fig. 5 A vs. C, D). The poor GB-CBM performances in warm conditions (CT and PR) suggest that additional heat stress modules are needed. The poor GB-CBM performance in ND suggests that additional day length or day length and temperature interaction terms are needed. The reason for the high bias can be explained by the NAR module and was discussed previously but is more prominent in the warmer sites.

**Fig. 6:**
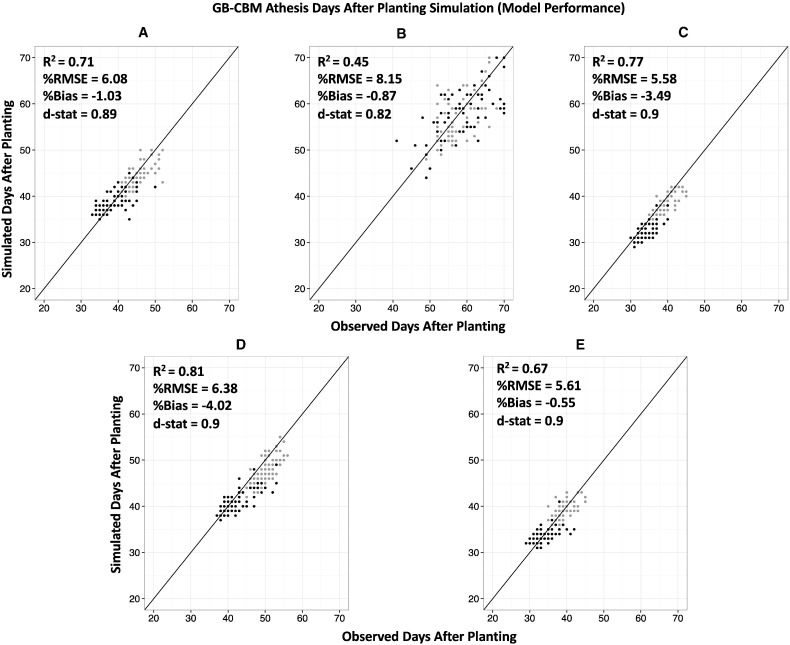
Simulated versus observed plots of Anthesis using the GB-CBM for 187 genotypes (RILs) by field sites (Citra (A), North Dakota (B), Palmira (C), Popayan (D), Puerto Rico (E)) with R^2^, %RMSE, %Bias, and Willmot agreement index. Determinate genotypes are black, while indeterminate genotypes are grey. The parameters were estimated across all sites but plotted to compare performance at each site. The lines represent the 1:1 relationships.

The GB-CBM simulated anthesis days after planting (ADAP) fairly well, except in the case of ND, with an average across sites R^2^, %RMSE, %Bias, and Willmot agreement index values of 0.68, 6.4, − 1.99, and 0.88, respectively (Fig. 6). The model did a relatively poorer job of capturing ADAP in ND (R^2^ = 0.45) compared to the other sites (Fig. 6), and is likely due to the fact that ND was the only site with long days and limited the accuracy in identifying long-day effects for the RF module (Fig. 6 B). The RF module appears to require additional adjustment in the low temperature responses for ADAP since there was some bias (− 4.02%) in the prediction of ADAP for the coolest site, Popayan (PO; Fig. 6 D).

## 4 Discussion

Several approaches to develop gene-based crop models with varying degrees of complexity have been suggested (White and Hoogenboom, 2003). The least complicated approach is to incorporate additive and epistatic effects as linear models that predict traditional GSPs (genetic coefficients) into crop models, provided data are available for a wide range of genotypes grown under a wider range of environments. A similar approach was proposed for the dry bean (*P. vulgaris L.*) simulation model in DSSAT (CROPGRO-Bean) in which seven genes that were assumed to affect phenology, growth habit, and seed size were used to predict GSPs based on linear functions of the genes, and the GSPs were used with the original functions for computing process rates (White and Hoogenboom, 1996). This gene-based model accurately predicted phenology but was unable to accurately predict yield variations (Hoogenboom et al., 1997). Integration of genetic information into other crops such as soybean (*Glycine* max *L*.; (Messina et al., 2006)), maize (*Zea mays* L.; (Reymond et al., 2003, Reymond, 2004, Chenu et al., 2009)), rice (*Oryza sativa*; (Gu et al., 2014)), and barley (*Hordeum vulgare* L.; (Yin et al., 2000a, Yin et al., 2000b, Yin et al., 2004)) resulted in varying degrees of success. For example, GSPs in the CROPGRO-Soybean model were converted into mathematical functions of the (day length-sensitive) *E* loci and used to simulate the reproductive development behavior of soybean cultivars. The modified model accounted for 75% of the variance in maturity date in independent cultivars from Illinois, USA based on weather data and 4 of the 6 *E* loci, which were found using SSR-linked markers (Messina et al., 2006). Chenu et al. (2009) modified the APSIM maize model (Keating et al., 2003) to allow parameters for leaf and silk elongation to be affected by QTL interactions to simulate maize growth under drought conditions. The study was able to construct a QTL network affecting the examined traits, and identify the best combination of traits for yield under the management practices in the experiment. Technow et al. (2015) recently demonstrated the utility of integrating a maize model with approximate Bayesian computation (ABC) algorithm for G and G × E effects to improve genomic prediction. Although the study used synthetic data, the ABC algorithm improved the maize model prediction accuracies relative to using statistical relationships based on markers alone.

More complex approaches for integrating genetics with crop models have been suggested and include simulating gene expression over the course of plant development or including polypeptide translation information (White and Hoogenboom, 2003). The higher level of biological networks/levels may increase errors within the models, and it has been argued that further increasing the level of complexity in regards to the genetics may not be necessary for further improving crop models as a breeding tool if they capture the physiological basis of the traits (Yin et al., 2004, Hammer et al., 2010). The approach suggested here would allow flexibility in designing modules at the desired level of biological complexity (also discussed in Yin et al., 2004). New gene-based modules can be built to replace sets of calculations for specific crop growth or developmental processes already in the existing CROPGRO-Bean model. These granular modules would be designed to incorporate G, E, and G × E factors to improve model capabilities to simulate performance of multiple genotypes across a range of environments. These modified models would have the capacity to quantify crop performance when new cultivars are developed or to test existing ones in target environments without having to conduct costly multi-location experiments.

The early stages of plant development are just one part of the growth and development processes of plants but they are important processes that also affect yield. In common bean genotypes with a determinate growth habit, the terminal meristem makes a transition from the vegetative to the reproductive phase thus ending the addition of nodes on the main stem (Ojehomon and Morgan, 1969). In indeterminate common bean genotypes, nodes continue to be added on the main stem after the reproductive phase has begun and this continues (Kwak et al., 2012) until achieving some maximal node number. The rate of node/leaf addition depends on temperature, the genotype, and CO_2_ levels (Reddy et al., 1995, Vallejos and Pearcy, 1987) and is associated with levels of miR156, squamosal-like proteins and cytochrome P450 genes in Arabidopsis (Schwarz et al., 2008, Wang et al., 2008). Node addition rate and the rate of progress toward flowering were both found to be under genetic and environmental control for the bean RILs used in these studies (Zhang and Developing, 2015, Bhakta, 2015).

We have constructed a prototype gene-based Common Bean Model (GB-CBM) to simulate early vegetative and reproductive development by integrating dynamic QTL effect models for node addition rate, rate of progression to anthesis, and the maximal main stem node number. The focus of this work was to illustrate an approach for transforming QTL effect models of growth and developmental processes into modular dynamic models for predicting early growth and development based on QTL, E, and QTL × E factors previously identified by linear mixed effect statistical models. The component models in this study were based on assumptions that the QTLs, E, and QTL × E are the same factors affecting trait development. For this reason, dynamic simulations of these traits can be accomplished through daily time steps using the daily values of the relevant environmental factors. As a result, the highly influential effects were captured with this approach while maintaining simplicity.

The prototype GB-CBM presented here could be expanded at different levels. First, additional modules for the growth of the main stem could include internode and leaf expansion rates, and rate of addition of branches. Also, the expansion of the GB-CBM for more complex traits that additionally affect yield will require modules for other processes (e.g., photosynthesis, leaf area expansion, seed and pod growth, senescence among others). Second, additional studies are needed to expand the identity of QTLs that had significant effects on the modeled traits. Further work with diversity panels (more genotypes), with additional environments could identify other QTLs and better estimate their interacting effects. Finally, the assumption of linearity of environment effects on a trait is another limitation in the current GB-CBM version since most biological processes have nonlinear responses to the environment. For example, many developmental and growth responses including node addition and flowering show a temperature response that is a piecewise function of base and optimal temperatures, such as used in the CROPGRO-Bean model (Jones et al., 2003). Other approaches to account for non-linear QTL effects over the growth cycle have been developed where logistic leaf senescence curve parameters for potato were directly predicted with QTL information with non-linear extension of mixed effect models (Malosetti et al., 2006). Here, the model did a poor job of simulating node development for the extreme temperatures (cold in PO and hot in CT), so incorporating nonlinear temperature functions for describing the dynamic processes is likely to improve predictions. This would be similar to functional mapping that targets the genes that control growth and development by treating these biological processes as nonlinear dynamic traits rather than static phenotypes (Ma et al., 2002, Wu et al., 2003, Malosetti et al., 2006, Wu and Lin, 2006, Yang et al., 2009). Functional mapping identifies and estimates the effect of dynamic QTLs by testing whether these parameters display significant differences among genotypes (Wu and Lin, 2006). Incorporating functional mapping approaches may lead to models that better represent the underlying biological responses of dynamic traits. Management (M) factors such as plant density, water, and nutrients were not included in the presented model. Conceivably, one could extend the approach to include M effects and G × E × M interactions, if phenotype in M conditions were included.

The dynamic processes modeled in this study (NAR(*t*) and RF(*t*)) are also included in the existing CROPGRO-Bean crop model. This is one reason that these two processes were selected. However, the functional relationships used in the existing bean model are very different from the equations developed by the dynamic QTL effect approach used in this paper (Eqs. (10), (12) for RF(*t*) and NAR(*t*), respectively). These differences deserve attention here. In the CROPGRO-Bean model, neither of these processes was dependent on genes/QTLs as they are in the approach described in this paper. In fact, NAR(*t*) (similar to the TRIFL parameter in the CROPGRO-Bean model) was assumed to have the same cardinal temperature dependency for all genotypes. Temperature was the only environmental variable used to compute daily node addition rates; this function is piece-wise linear (using hourly temperatures) with a base temperature of 5 °C, an optimum temperature of 27 °C above which further increases in temperature do not increase node appearance rates, and two other temperature thresholds that describe a slowing rate (above 37 °C) and no development above 45 °C. These two upper thresholds are highly uncertain. Eq. (12) includes daily mean temperature, daily solar radiation, day length and four QTLs and Fig. A.2 (supplemental material) shows the temperature effect developed from data in this study to be linear, with a base temperature somewhat lower than 10 °C that appears to vary among genotypes. The extent that these cardinal temperatures are affected by genotype needs to be explored since others propose these may not be genetically controlled for some species (Parent and Tardieu, 2012). Additional work is needed to evaluate whether incorporating nonlinear functions for environmental effects in the dynamic QTL effect models would improve results.

**Fig. A.1:**
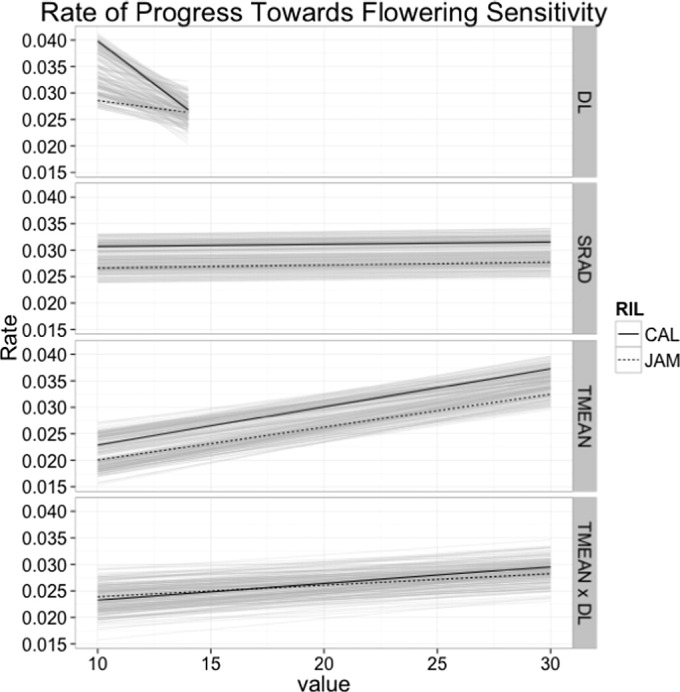
Environment sensitivity analyses for rate of progress toward flowering (RF) with parent lines highlighted. Grey lines represent the RI population. For each day length (DL) sensitivity analysis, mean temperature and solar radiation values were held at 21.35 °C and 18.31 **MJ m**^**− 2**^ d^− 1^, respectively. Simulated rate was then predicted with DL values from 10 to 14 h in increments of 0.5 h. For each solar radiation (SRAD) sensitivity analysis, mean temperature and day length values were held at 21.35 °C and 12.7 h, respectively. Simulated rate was then predicted with SRAD values from 10 to 30 **MJ m**^**− 2**^ d^− 1^ in increments of 0.5 **MJ m**^**− 2**^ d^− 1^. For each mean temperature (TMEAN) sensitivity analysis, solar radiation and day length values were held at 18.31 **MJ m**^**− 2**^ d^− 1^ and 12.7 h, respectively. Simulated rate was then predicted with TMEAN values from 10 to 30 °C in increments of 0.5 °C. For each temperature by day length interaction (TMEAN × DL) sensitivity analysis, solar radiation and day length values were held at 18.31 **MJ m**^**− 2**^ d^− 1^ and 14 h, respectively. Simulated rate was then predicted with TMEAN values from 10 to 30 °C in increments of 0.15 °C.

**Fig. A.2:**
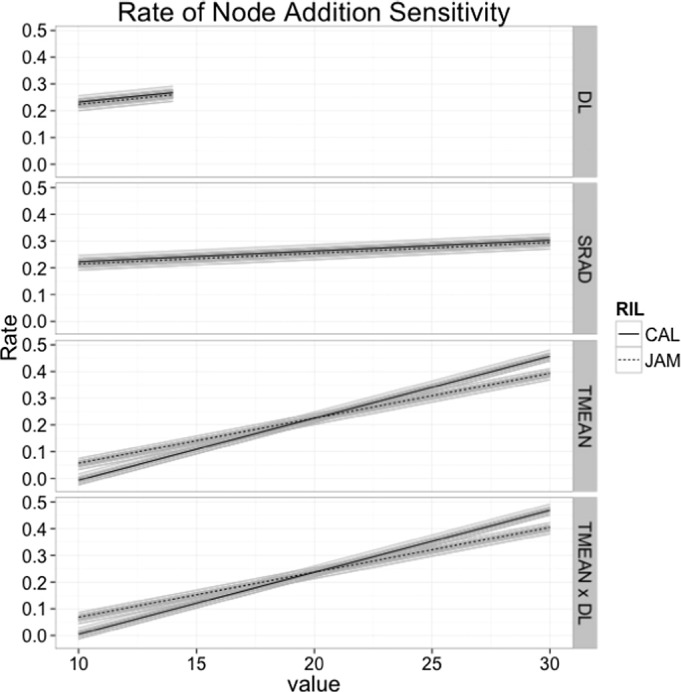
Environment sensitivity analyses for node addition rate (NAR) module with parent lines highlighted. Grey lines represent the RI population. For each day length (DL) sensitivity analysis, mean temperature and solar radiation values were held at 21.51 °C and 17.38 **MJ m**^**− 2**^ d^− 1^, respectively. Simulated rate was then predicted with DL values from 10 to 14 h in increments of 0.5 h. For each solar radiation (SRAD) sensitivity analysis, mean temperature and day length values were held at 21.51 °C and 12.74 h, respectively. Simulated rate was then predicted with SRAD values from 10 to 30 **MJ m**^**− 2**^ d^− 1^ in increments of 0.5 **MJ m**^**− 2**^ d^− 1^. For each mean temperature (TMEAN) sensitivity analysis, solar radiation and day length values were held at 17.38 **MJ m**^**− 2**^ d^− 1^ and 12.74 h, respectively. Simulated rate was then predicted with TMEAN values from 10 to 30 °C in increments of 0.5 degrees. For each temperature by day length interaction (TMEAN x DL) sensitivity analysis, solar radiation and day length values were held at 17.38 and 14 h, respectively. Simulated rate was then predicted with TMEAN values from 10 to 30 °C in increments of 0.15 degrees.

Comparison of the dynamic QLT effect model (Eq. (10)) with the process model used to predict daily rate of progress toward flowering, RF(*t*)), in the CROPGRO-Bean model is somewhat more complex. In the existing bean model, a multiplicative model formulation is used as follows (Eq. (13)):

(13) $$\mathrm{RF}\left(t\right)=\mathrm{MR}\cdot f\left(T_{\mathrm{hour}}\left(t\right)\right)\cdot g\left(P\left(t\right)\right)$$

where MR is a maximum daily rate that is a GSP (normally estimated from field data), *f(T*_*hour*_*(t))* is a nonlinear function of hourly temperature (*T*_*hour*_*(t)*) and assumed to be the same function for all genotypes, and *g(P(t))* is a nonlinear function of day length (Boote et al., 2013) with GSPs for critical day length (CSDL) and for sensitivity to increases in day length above the critical threshold (PPSEN). Comparing this equation with Eq. (10) shows that 12 QTLs affect RF(*t*), and of course the equation uses only linear responses to environmental factors and includes interactive terms for temperature and day length. Fig. A.1 shows that the dynamic QTL effect model produced similar responses to day length as had been assumed in the existing bean model, with the negative slope of RF(t) being analogous to PPSEN. However, the effect of day length depends on temperature in Eq. (10) due to the TMEAN(*t*)·DL(*t*) term in the equation, and these effects vary among genotypes. This figure also shows that the relationship with temperature varies with genotype, which had not been considered in the existing bean model.

Based on these results, one may suggest that process models be developed using dynamic QTL effect methods, using prior knowledge of the functional relationships that crop modelers have demonstrated in specific physiological studies (as demonstrated here). For example, one could implement Eq. (10) in place of existing computations in the CROPGRO-Bean model for flowering time simulations. One could even introduce hourly temperatures, similar to those used in current crop models. This would result in equations that would be very different from Eq. (13), and look more like Eq. (10) but use nonlinear instead of linear terms. An alternative to this approach would be to use the linear mixed effect models that can be used by researchers who are exploring G, E, M, and G × E × M interactions. Then, crop modelers could use that information to revise their original modules for different processes as more information is developed, making the terms in the functions that they use, similar to Eq. (13), depend on genes and G × E interactions. For example, T_b_ and T_opt_ values for the temperature function in Eq. (13) can be predicted with identified G, E, and G × E factors. We believe that both of these approaches have merit and should be pursued. One advantage of expanding mixed effect models to model dynamic processes is that software could be created to develop nonlinear mixed effect models of dynamic processes and this could encourage more involvement of geneticists and bioinformaticians in gene-based crop modeling.

One of the major implications of this study is the critical need for phenotyping data that have wide variations in genetic characteristics combined with wide variations in environmental variables (including temperature and day length). In fact, for this study there were only five environments, which limited the reliability and inference of some of the parameters estimated relative to sensitivities to E and G × E. True model evaluation will require that new field sites that were not used in the calibration process be validated. One promising approach might be to make further use of the many yield trials that are conducted by plant breeders in different states and regions. Additionally, automated phenotyping techniques will improve the feasibility of these efforts. By combining genotype information with phenotypic information, it may be possible to make rapid advances toward more holistic, gene-based crop development, growth, and yield models.

## 5 Conclusion

Although traditional crop models are able to reproduce some G × E interactive effects on yield through GSPs, they have not adequately represented G × E interactive effects at the level of dynamic growth and development processes. The approach demonstrated here incorporates these important interactions at a process level, which are likely to enrich these G × E interaction effects on yield. Empirical GSPs have to be estimated for every genotype, which is costly and time consuming. We showed that there is potential for quantifying rates of vegetative and reproductive development of crops with dynamic QTL effect models based on G and E information identified from mixed effect statistical approaches, using data from multiple locations, and that this could eliminate the need for one to conduct experiments in multiple locations when a new cultivar is released, if the alleles of pertinent genes of the cultivars are known. Nonlinear dynamic QTL effect models are needed to establish a functional relationship between a given trait and the genetic and environmental factors that contribute to the trait along with the G × E interactions; the dynamics of these factors will provide a better representation of the biology of any growth or developmental process. Although the approach used to model vegetative and reproductive development processes in this study was successful, it is not yet clear whether this approach can be used to develop component modules of other growth processes, such as dry matter growth and partitioning to grain yield. Next generation crop models can be built through the type of incremental improvements as described here. In the future, it should be possible for one to genotype a new cultivar and be able to predict crop performance in a range of environments with good accuracy.

The following are the supplementary data related to this article.

Table A.1 Weather file for the five sites: (Citra, FL (CT); Palmira, Colombia (PA); Popayan, Colombia (PO); Isabela Puerto Rico (PR); and Prosper, North Dakota (ND)) Table A.1.

Table A.2 Molecular markers (QTL) used in the GB-CBM with RF, MSNOD_max, and NAR dynamic QTL effect models Table A.2.

## Figures and Tables

**Table A.3:** The significant terms in the linear mixed effect model for maximum number of nodes along the main stem (MSNODmax) module.

Significant Term	Chi Pr.^b^
Mean MSNOD_max_	–
TMEAN^a^	***
SRAD^a^	***
DL^a^	***
MSN1	***
MSN2	**
MSN3	***
MSN4	***
MSN5	***
MSN6	0.134^c^
MSN2 × TMEAN^a^	***
MSN2 × SRAD^a^	***
MSN2 × DL^a^	***
MSN6 × TMEAN^a^	⁎⁎, c
MSN6 × SRAD^a^	***

a Mean values across sites for TMEAN[°C]: SRAD[MJ·d^− 1^]: DL[*hr*] are 21.85: 18.74: 12.81, respectively

b Chi-square probabilities for each term from the mixed effect model analysis

⁎⁎⁎ denotes chi-square probability with 1df < 0.001.

⁎⁎ denotes chi-square probability with 1df < 0.01.

c MSN6 remained in the model because MSN6 × TMEAN was found to be significant in the final model
